# Effects of organic and chemical nitrogen fertilization and postharvest treatments on the visual and nutritional quality of fresh‐cut celery (*Apium graveolens* L.) during storage

**DOI:** 10.1002/fsn3.3063

**Published:** 2022-10-01

**Authors:** Mesbah Babalar, Hadiseh Daneshvar, Juan Carlos Díaz‐Pérez, Savithri Nambeesan, Leila Tabrizi, Mojtaba Delshad

**Affiliations:** ^1^ Department of Horticultural Sciences, Faculty of Agricultural Science and Engineering, College of Agriculture and Natural Resources University of Tehran Karaj Iran; ^2^ Department of Horticulture College of Agricultural and Environmental Sciences University of Georgia Tifton Georgia USA; ^3^ Department of Horticulture College of Agricultural and Environmental Sciences University of Georgia Athens Georgia USA

**Keywords:** blanching, cold storage, edible coating, precooling, safety

## Abstract

The shelf life of horticultural commodities depends on pre‐ and postharvest factors, such as soil fertilization and postharvest handling. The current study aimed to evaluate fresh‐cut celery's postharvest quality as affected by the rate and type (organic and chemical) of nitrogen (N) fertilizer and postharvest treatments. Celery (‘Tall Utah’) crop was grown in a field in Karaj, Iran. The experimental design was a randomized complete block with three replications and seven preharvest (fertilizer), and five postharvest treatments. Organic fertilizers were vermicompost (VER) and bio‐organic fertilizer [farmyard and livestock manure plus *Trichoderma harzianum* (COM)]. Chemical fertilizers were urea (46% N) at high rate [322 kg·ha^1^ N (UREA_HIGH)], optimal rate [196 kg·ha^−1^ N (UREA_OPT)], and low rate [138 kg·ha^−1^ N (UREA_LOW)]; ammonium nitrate [35% N (AN)] at 196 kg·ha^−1^ N; and treatment without fertilization was used as a control. Postharvest treatments included plastic packaging (PP), hydrocooling (HC), blanching (B), and edible coating of psyllium seed mucilage (EC). After postharvest treatments, celery petioles were stored (0–2°C, 85%–90% RH) for 4 weeks and evaluated weekly for quality attributes. Organic fertilizers and UREA_LOW were the most effective treatments in reducing the changes in color, weight loss, titratable acidity (TA), pH, and total soluble solids (TSS) of fresh‐cut celery. Organic fertilizers enhanced the vitamin C content, total phenols, and antioxidant activity in celeries. As postharvest treatments, hydrocooling, plastic packaging, and blanching maintained chroma and hue values. Blanching had the greatest effect on the L* value. Hydrocooling increased celery's TA, TSS, and vitamin C content and reduced weight loss and pH during storage. Thus, celery quality was improved when grown under low or adequate N fertilization. Hydrocooling was an effective postharvest treatment for preserving fresh‐cut celery quality during storage.

## Graphical abstract

Nowadays, consumers have an increasing preference for vegetables produced using healthier and environmentally friendly techniques. The postharvest quality of fresh‐cut celery is affected by nitrogen (N) fertilizer rate and type (organic and chemical) as well as postharvest treatments. In the case of postharvest treatments, we applied physical and eco‐friendly methods including precooling, blanching, and edible coating of psyllium seed mucilage. The results showed that phytochemical compounds were enhanced with organic fertilizers and the high fertilizer rate was associated with low celery quality. Additionally, Hydrocooling had the most effect on the nutritional celery quality.

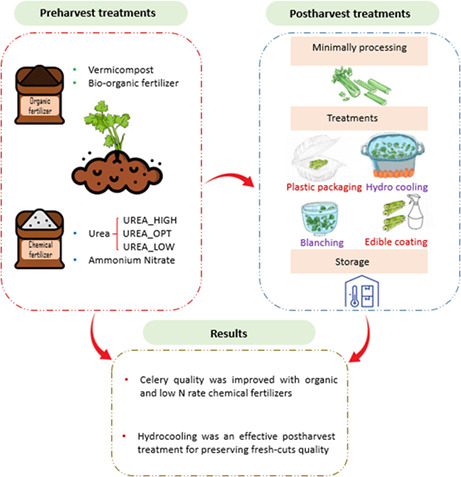

## 1 INTRODUCTION

Celery (*Apium graveolens* dulce) is a plant from the Apiaceae family that is appreciated in the diet for its reduced caloric content and high nutritional value (Sowbhagya et al., 2010). Dried celery is used as a spice, and fresh celery petioles are mainly consumed in salads. Celery is compatible with minimal processing and is usually available in the market as fresh cut.

Postharvest losses of crops may be affected by preharvest factors, harvesting, and postharvest operations such as precooling, blanching, sorting, grading, packaging, transportation, and storage (Adhikary & Kumar, 2021). Preharvest factors such as genotype, weather conditions, fertilization, and cultural operations affect produce quality (Kader, 2008). Celery yield, mineral content, and quality are affected by the amount, frequency, and method of soil fertilization. Nitrogen (N) is an essential nutrient applied as organic or inorganic fertilizers (Zhang et al., 2019). Excessive N fertilization may cause a high nitrate accumulation in crops, negatively affecting the safety, nutritional quality, and shelf‐life of raw and minimally processed vegetables (Cui et al., 2020; Miceli & Miceli, 2014). Therefore, fertilization must be carefully managed to minimize crop nitrate accumulation and maximize crop yield and environmental protection (Saleh et al., 2019).

The undesirable effects of chemical fertilizers on human and environmental health could be minimized by organic fertilizers (Abou‐El‐Hassan et al., 2017; Naeem et al., 2006), such as vermicompost and biofertilizers (organic fertilizers with beneficial soil microorganisms). Vermicompost is produced via a biodegradation technique of organic waste using earthworms and microorganisms (Shishehbor et al., 2013). It has various benefits, which include soil particle stabilization, enhanced soil fertility, and the recovery of nutrients. In addition, vermicompost provides plant growth hormones such as gibberellins, auxins, and cytokinins (Ravindran et al., 2015; Singh et al., 2020; Song et al., 2015). Composts can be considered an eco‐friendly alternative for plant mineral nutrition. Composts increase soil water retention capacity and soil organic matter content (Mehta et al., 2014; Ning et al., 2017; Shen et al., 2013). Composts are used for biofertilizer preparation because of their physical and chemical properties. Composts supply macro‐ and micronutrients, antibiotics, and plant growth‐regulating substances (Kumar et al., 2017; Naveed et al., 2015).

Precooling removes the field heat from freshly harvested produce and is one of the most effective physical methods in slowing biological processes (Ambaw et al., 2017; Hardenburg et al., 1986; Zhu et al., 2019). Commonly precooling methods include forced air cooling, hydrocooling, liquid ice cooling, and vacuum cooling (Hardenburg et al., 1986). Hydrocooling uses chilled water to remove heat from fresh produce. Due to water's high heat transfer coefficient, it is an effective cooling technique for many fruits and vegetables (Becker & Fricke, 2002; Liang et al., 2013). The present study assessed the effect of hydrocooling on some postharvest characteristics of celery grown under organic and chemical nitrogen fertilization.

In recent years, rapid industrialization, population growth, and changed lifestyles increased the demand for processed and ready‐to‐eat packed foods. Packaging plays a crucial role in enhancing the postharvest life of the product. The ideal packing material should be cost‐effective, readily available, easy to handle, and environmentally friendly, with low weight and adequate ventilation. Desirable packaging attracts the consumer to buy the product and significantly reduces the risks during shipping (Adhikary & Kumar, 2021). Types of packaging materials include plastics, cane baskets, wooden boxes, clay vessels, metal cans, China pots, and paper bags (Adhikary & Kumar, 2021; Watson et al., 2015). Plastics are the most wide‐ranging and ordinary materials to store foods, salads, and fresh‐cut vegetables and fruits at local markets or for retail purposes (Adhikary & Kumar, 2021). Increased use of synthetic packing material is considered an environmental threat; hence, some coating techniques evolved to prolong produce's shelf life and reduce plastic waste (Adhikary & Kumar, 2021; Marsh & Bugusu, 2007; Zhao et al., 2019). Edible coatings and films, known as eco‐friendly packaging, could potentially replace traditional polymer packaging (Bashir et al., 2017; Campos‐Requena et al., 2015; Jaramillo et al., 2016; Moghadam et al., 2020). Biodegradable films and edible coating are used to achieve optimal exchange of moisture, gases, flavors, and lipids, as well as serving as a carrier for additives such as antioxidants, antimicrobials, flavoring, and coloring (Bourtoom, 2008; Falguera et al., 2011; Fu & Dudley, 2021; Song et al., 2011).

Blanching (hot water dip) is a disinfectant method for minimally processed horticultural crops. This method exposes the product to high temperatures for a short period. Blanching decreases degradation reactions, deactivates enzyme activities, and maintains product color. Blanching also reduces decay, browning, and chilling injury in subtropical crops during low‐temperature storage (Arroqui et al., 2003; De Corato, 2019; Rehman et al., 2021; Rico et al., 2007).

Our study aimed to investigate the effects of N organic and chemical fertilizers on postharvest quality and the impact of postharvest treatments on the safety and quality of fresh‐cut celery.

## 2 MATERIALS AND METHODS

### 2.1 Plant material

The study was conducted at the Research Center of the Horticultural Sciences Department, University of Tehran, Karaj, Iran. The mean annual temperature and cumulative rainfall were 25.6°C and 10.1 mm during the experiment. The soil's chemical properties (0–30 cm depth) were analyzed according to standard laboratory procedures (Klute & Page, 1986). The soil pH was 8.02, soil electrical conductivity 3.47 ds·m^−1^, organic matter 1.68%, total nitrogen 0.18%, available phosphorus 62.87 mg·kg^−1^, available potassium 338.24 mg·kg^−1^, available calcium 463.4 mg·kg^−1^, and available magnesium 82.8 mg·kg^−1^. Celery seedlings (‘Tall Utah’) were grown in a greenhouse and transplanted to the field on May 30.

The experimental design was a randomized complete block with three replications. There were seven preharvest and five postharvest treatments. Each field plot (replicate) was 8 m × 3.6 m and had 48 seedlings. The distance between plots was 0.8 m. The spacing between rows and plants within each plot was 0.4 and 0.3 m, respectively. Drip irrigation and manual weeding were performed as necessary (about every 2 days). Plants were harvested on Oct 14 after reaching marketable size, when the majority of plants had reached a height of 40–45 cm and the base of the plants was 5–7 cm thick. Ten plants from the inner part of each plot were randomly collected and transferred to the laboratory for postharvest treatments.

### 2.2 Preharvest treatments (N fertilization)

Seven fertilization treatments were evaluated: two organic fertilizers [vermicompost (VER; 196 kg·ha^−1^ N) and a bio‐organic fertilizer (COM; 196 kg·ha^−1^ N)]; four chemical fertilizers, urea (46% N) at three different rates [UREA_HIGH (322 kg·ha^−1^ N), UREA_OPT (196 kg·ha^−1^ N), and UREA_LOW (138 kg·ha^−1^ N)]; ammonium nitrate (AN; 35% N at 196 kg·ha^−1^ N); and one nonfertilized treatment (control). The recommended N‐fertilizer rate for local celery growers was 196 kg·ha^−1^ N. The bio‐organic fertilizer (COM) was composed of a mixture of the composted farmyard and livestock manure with *Trichoderma harzianum* (5% w/v ratio). The mixture was maintained at room conditions (25°C, 50% RH) for 5 days and manually stirred daily. The *T. harzianum* strain was prepared from the Soil Science Laboratory. The isolate was cultured on potato dextrose agar (PDA) medium and kept in an incubator at 28°C for 7 days. Five samples from each fungi colony were inoculated into 100 ml of the potato dextrose (PD) medium and, to obtain the spore suspensions, kept for 3 days in a shaking incubator (250 rpm·min^−1^) at 28°C. Organic fertilizers were applied 7 days before planting as a one‐time application. Chemical fertilizers were applied as a top dressing (three times) with irrigation at intervals of 3–4 weeks during the growing season. The first application was in week 1 after planting the seedlings. Before planting, the chemical properties of organic fertilizers were determined (Table 1).

**TABLE 1:** Chemical analysis of the organic fertilizers used for the experiment.

Organic fertilizer	N	P	P_2_O_5_	K	K_2_O	EC
	%					*ds*/*m*
VER	0.76	0.45	1.03	0.11	0.13	1.2
COM	1.56	0.16	0.37	0.38	0.46	6.27

Abbreviations: COM, compost + *Trichoderma harzianum* (196 kg·ha^−1^ N); VER, vermicompost (196 kg·ha^−1^ N).

### 2.3 Minimally processing

Roots, leaves, and basal parts of rosettes were removed to obtain the unbranched petioles. The petioles were washed with distilled water, air‐dried, and sliced into 12‐cm‐long pieces using a sharp stainless‐steel knife. Petioles with a similar visual appearance and free of defects (decay, insect, or handling damage, discoloration, and dehydration) were minimally processed.

### 2.4 Postharvest treatments

Postharvest treatments were evaluated for each replicate and fertilizer treatment. The postharvest treatments were as follows: control (no postharvest treatment and packaging), plastic packaging (PP), hydrocooling (HC), blanching (B), and edible coating of psyllium seed mucilage (EC). Each replicate consisted of about 300 g of fresh‐cut celery.

The PP treatment was performed by packaging fresh‐cut celery into clear plastic square‐hinged containers. Hydrocooling was applied by immersing the celery in a small tank filled with about 180 L of a mixture of water and crushed ice (0–1°C) until the temperature of the celery reached a constant value. Celery was placed into a small stainless‐steel wire mesh basket for adequate water circulation. During hydrocooling, celery temperature was measured using a digital thermocouple equipped with a steel probe (Kochhar & Kumar, 2015). At the end of hydrocooling, the excess water of the samples was drained before packaging.

For the edible coating treatment, the psyllium seeds were carefully first cleaned by sieving to remove stones, chaff, and foreign matter. Then, the mucilage was extracted with a 1:30 ratio of seed to distilled water. The mixture was heated at 40°C for 1 h using an adjustable heater stirrer. Next, mucilage was filtered (by a cheesecloth) and oven dried at 50°C for 24 h. Two grams of the powdered psyllium seed mucilage was gradually added to 100 ml of distilled water and stirred for homogenization at 40°C. The fresh‐cut celeries were immersed in the coating solution for 2 min. After draining (to remove the coating solution excess), celeries were placed at room temperature for 20 min to dry (Banasaz et al., 2013; Yousuf & Srivastava, 2015). Blanching treatment was applied by dipping the celeries in hot water for 30 s at 70°C. In order to stop the cooking process and reduce heat, the samples were submerged in an ice‐water bath and drained.

### 2.5 Packaging and storage condition

Each replicate and treatment consisted of a 300 g sample of fresh‐cut celery. All treatments except the control and EC were packaged in clear plastic square‐hinged containers similar to PP treatment packaging. The control and EC were placed in the open air. All treatments were stored (0–2°C, 85%–90% RH) for 4 weeks and celery's quality attributes were evaluated weekly.

### 2.6 Petiole color (chroma, hue, and L*)

The color of fresh‐cut celery was measured based on McGuire (1992) using a colorimeter (Konica Minolta CR‐403). Three petioles from each treatment and replicate were randomly selected, and the color of the petiole's outer surface was measured. The color was determined as L*, a*, and b* values. L* indicates lightness, and hue angle and chroma were estimated from a* and b* values.

(1) $$\mathrm{Hue}=\tan^{-1}\left(\frac{b^{*}}{a^{*}}\right)\;\text{when}\;a^{*}>0\;\text{and}\;b^{*}>0$$

(2) $$\mathrm{Hue}=180{}^{\circ}+\tan^{-1}\left(\frac{b^{*}}{a^{*}}\right)\;\text{when}\;a^{*}<0\;\text{and}\;b^{*}>0$$

(3) $$\text{Chroma}={\left[{\left(a^{*}\right)}^{2}+{\left(b^{*}\right)}^{2}\right]}^{1/2}$$

### 2.7 Weight loss

Fresh‐cut petioles were weighed before applying postharvest treatments in each replicate and treatment, giving the initial petiole weight. The petiole weight loss was calculated as the difference between the initial and final weight of the fresh‐cut petiole during storage. Weight losses were calculated with the following equation:

(4) $$\text{Weight loss}\;\left(\%\right)=\frac{\text{Initial weight}-\text{Final weight}}{\text{Initial weight}\;}\times 100$$

### 2.8 Titratable acidity (TA), pH, and total soluble solids (TSS)

The juice of fresh‐cut celery from each replicate and treatment was used to determine TA, pH, and TSS. The TA was determined based on titration with 0.1 N NaOH until we reached 8.2 pH. The pH of the juice was recorded using a pH meter (Jenway 3320). The TSS content of celery juice was measured by a handheld refractometer (Atago N1) at room temperature.

### 2.9 Vitamin C

Vitamin C content for each replicate and treatment was measured with the titration method. The samples were titrated with iodine (I) and potassium iodide (KI) (16 g KI and 1.72 g I in 1 L water) solution. Starch (2%) was used as an indicator and added until the color changed to dark blue and was stable for a few seconds (O'Grady et al., 2014). The vitamin C content was calculated according to the following equation.

(5) $$\text{Vitamin}\;C=\frac{0.88\;\times V}{5}\times 100\;$$

*V* is the volume of the consumed I + KI solution.

### 2.10 Total phenol (TP) and antioxidant activity (AA)

A 1‐g frozen sample from each replicate and treatment was homogenized with 5 ml of 85% methanol solution. The suspension was centrifuged at 16,000 *g* for 10 min at 4°C. The supernatant was collected and used for TP and AA analysis. The TP was measured using the Folin–Ciocalteu reagent method (Singleton & Rossi, 1965). Briefly, 0.2 ml of the suspension was mixed with 1:10 diluted Folin–Ciocalteu and allowed to stand for 10 min. Then, 0.8 ml of sodium carbonate solution (7% w/v) was added and kept at 25°C for 30 min. The absorbance was measured at 760 nm, and gallic acid was used as the calibration standard. The results were expressed as mg of GAE [gallic acid equivalents per 100 g of FW (fresh weight)]. DPPH assay (2,2‐diphenyl‐1‐picryldrazyl) was performed to measure total antioxidant activity (AA) based on the method of Brand‐Williams et al. (1995) with slight modification. The working solution was prepared by making a methanolic solution of DPPH (0.1 mM). A 75 μl of methanolic extract was added to DPPH and kept in the dark for 30 min at 25°C. Absorbance was recorded at 760 nm using a spectrophotometer.

### 2.11 Polyphenol oxidase activity (PPO) and protein content assay

The PPO extraction and assay for each replicate and treatment were according to previous methods (Kar & Mishra, 1976; Zhan et al., 2009), with a few modifications. The PPO enzyme was extracted by mixing 1 g of frozen sample with 50 mM potassium phosphate buffer (pH = 7.8), followed by centrifugation at 16,000 *g* for 15 min at 4°C. The reaction solution contained 3 ml of 50 mM potassium phosphate buffer (pH = 7), 300 mM catechol solution, and 50 μl of the enzyme extract. Absorbance was recorded at 480 nm wavelength for 3 min. The total protein was assayed by the method of Bradford (1976), using bovine serum albumin (BSA) as a standard.

### 2.12 Statistical analysis

The experiment consisted of three factors (fertilization, postharvest, and storage). The field experiment was conducted as a randomized completed block design with three replications. The five postharvest treatments were applied to celeries from the seven fertilization treatments. The fertilizer × postharvest combinations were evaluated weekly during the 4‐week storage. Two‐way ANOVA was performed when the variables were measured during storage. Duncan's multiple‐range test was performed to determine significant differences among fertilizer treatments, postharvest treatments, and storage time. All statistical analyses were carried out with the ANOVA procedure of SAS 9.4 (Statistical Analysis System). Figures were drawn with R software (v4.0.3).

## 3 RESULTS

### 3.1 Petiole color (chroma, hue, and L*)

**FIGURE 1:**
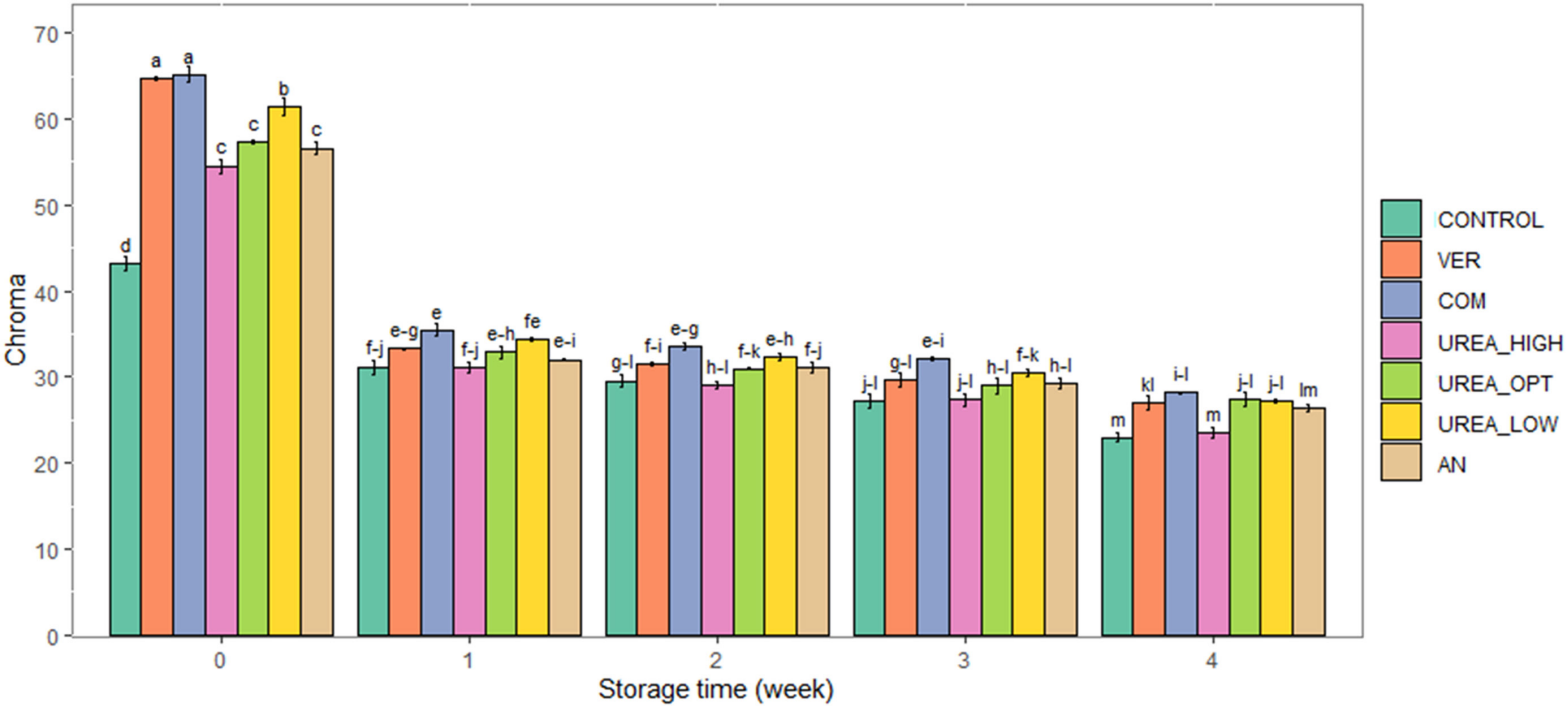
The interaction effect of storage time with nitrogen fertilizers (organic and chemical) on changes in chroma in fresh‐cut celery during the storage (0–2°C, 85%–90% RH) for 4 weeks. AN, 560 kg ammonium nitrate ha^−1^ (196 kg·ha^−1^ N); COM, compost + *Trichoderma harzianum* (196 kg·ha^−1^ N); CONTROL, no fertilizer; UREA_HIGH, 700 kg urea ha^−1^ (324 kg·ha^−1^ N); UREA_LOW, 300 kg urea ha^−1^ (139 kg·ha^−1^ N); UREA_OPT, 423 kg urea ha^−1^ (196 kg·ha^−1^ N); VER, vermicompost (196 kg·ha^−1^ N). Data are represented as mean. The similar small letters indicate non‐significant difference at a 5% level of probability using Duncan's test.

**FIGURE 2:**
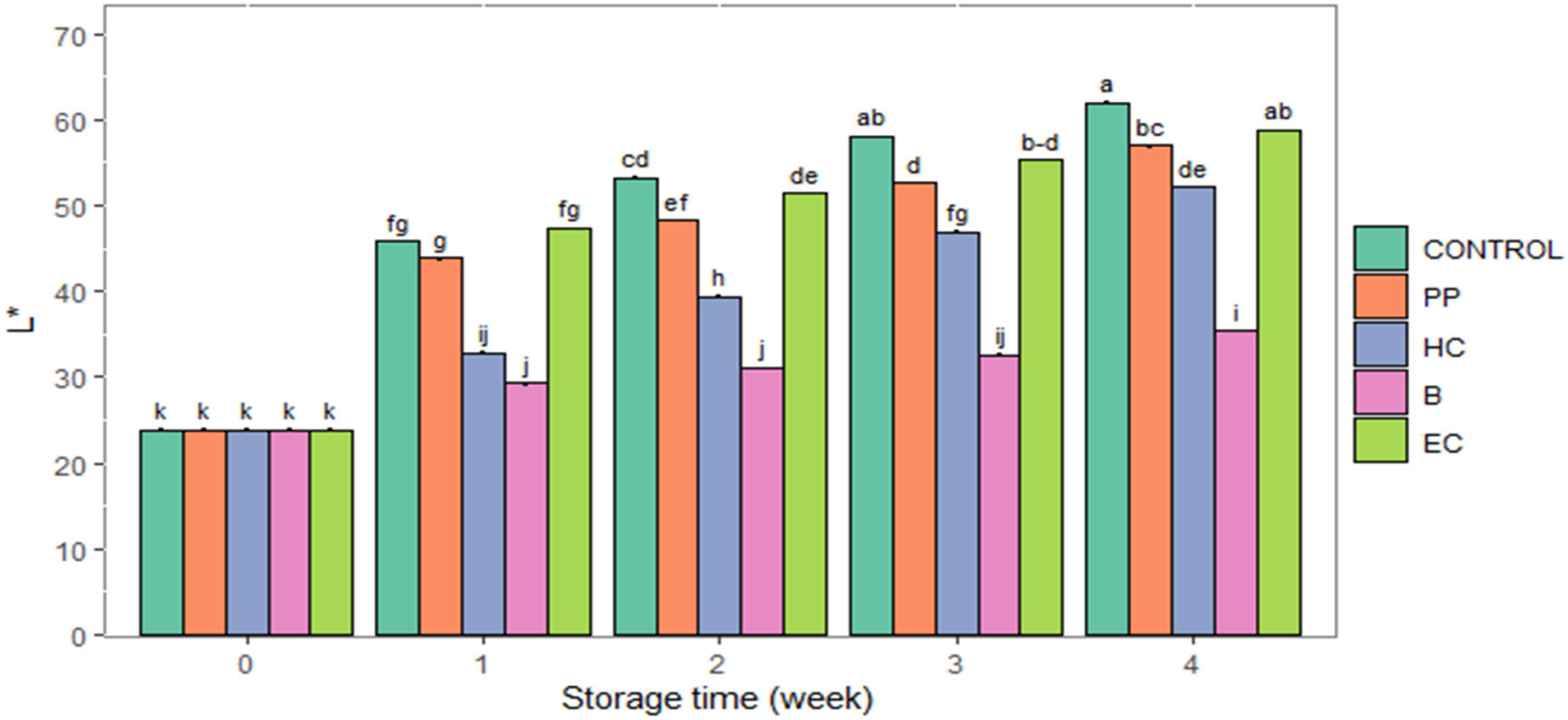
The interaction effect of storage time with postharvest treatments on changes of L* in fresh‐cut celery during the storage (0–2°C and 85%–90% RH) for 4 weeks. B, blanching in hot water; CONTROL, no postharvest treatment and packaging; EC, edible coating of psyllium seed mucilage; HC, hydrocooling; PP, plastic packaging. Data are represented as means. The similar small letters indicate non‐significant differences at a 5% level of probability using Duncan's test.

Storage time, fertilizer, and postharvest treatments significantly affected chroma, hue, and L* (*p* ≤ .01) in fresh‐cut celery. Interactions of storage time × fertilizer treatment were found to be significant for chroma (*p* ≤ .01) and storage time × postharvest treatment for L* value (*p* ≤ .01) (Table 2). Chroma value decreased during storage (Figure 1). At harvest time, the chroma value was the highest in organic fertilizers; later, there was no difference between treatments. Postharvest treatments maintained chroma values compared with the control (Table 2). The hue angle decreased during storage. Hue angle was the highest in COM, followed by VER, and was lowest in UREA_HIGH and control. Postharvest treatments resulted in higher hue values than the control (Table 2), with the greatest benefits exerted by PP, HC, and B treatments. The L* value increased during the storage (Figure 2) and was the lowest in the control and UREA_HIGH and highest in COM. All postharvest treatments reduced L* value changes during storage compared with the control; the lowest L* values were observed in the B treatment (Figure 2).

**TABLE 2:** Effect of storage time, nitrogen fertilizers (organic and chemical), and postharvest treatments on postharvest quality of fresh‐cut celery during storage (0–2°C, 85%–90% RH) for 4 weeks.

	Chroma	Hue	L*	Weight loss (%)	TA	pH	TSS (%)	Vit C content	TP (mg·GAL g^−1^·FW)	AA (%)	PPO (U·mg^−1^ protein·min^−1^)
Storage time (week)											
0	57.56^a^	118.42^a^	38.12^e^	0.00^e^	0.12^a^	6.16^e^	7.29^a^	7.54^a^	7.57^b^	78.50^b^	10.17^b^
1	32.88^b^	115.74^b^	44.53^ab^	4.05^d^	0.08^b^	6.34^d^	5.57^b^	7.10^b^	8.10^a^	82.57^a^	11.80^a^
2	31.12^c^	115.07^bc^	45.70^a^	5.94^c^	0.07^c^	6.42^c^	5.27^c^	6.65^c^	7.10^b^	76.25^b^	8.36^c^
3	29.29^d^	113.90^c^	39.51^cd^	8.28^b^	0.06^c^	6.47^b^	4.94^d^	6.06^d^	6.29^c^	69.22^c^	6.16^d^
4	26.06^e^	111.87^d^	41.27^cd^	10.87^a^	0.05^d^	6.57^a^	4.56^e^	5.40^e^	5.50^d^	62.80^d^	4.72^e^
Fertilizer treatment											
Control	30.80^e^	111.19^e^	38.12^e^	5.28^de^	0.05^c^	6.52^a^	5.03^c^	6.66^bc^	7.24^ab^	75.13^bc^	8.31
VER	37.21^b^	117.28^ab^	44.53^ab^	4.81^e^	0.09^a^	6.28^e^	6.02^a^	6.70^b^	7.57^a^	78.33^b^	8.18
COM	38.88^a^	118.43^a^	45.70^a^	5.14^de^	0.09^a^	6.35^d^	5.97^a^	7.50^a^	7.81^a^	84.70^a^	8.12
Urea_HIGH	33.08^d^	112.40^e^	39.51^de^	7.22^a^	0.07^b^	6.44^b^	5.21^c^	5.70^e^	6.08^d^	66.34^e^	8.50
Urea_OPT	35.50^c^	115^cd^	41.27^cd^	6.06^bc^	0.07^b^	6.39^bcd^	5.61^b^	6.09^de^	6.22^d^	68.33^e^	8.30
Urea_LOW	37.16^b^	116.39^bc^	43.24^bc^	5.75^cd^	0.08^b^	6.36^d^	5.63^a^	6.60^bc^	6.85^bc^	73.93^cd^	8.68
AN	35.05^d^	114.33^d^	42.34^c^	6.53^ab^	0.07^b^	6.42^bc^	5.03^c^	6.31^cd^	6.60^cd^	70.31^de^	8.52
Postharvest treatment											
Control	33.17^b^	112.17^c^	48.59^a^	21.12^a^	0.05^e^	6.47^a^	5.44^b^	6.15^b^	6.85	72.85	8.16
PP	35.88^a^	115.66^a^	45.11^b^	1.59^d^	0.08^b^	6.34^b^	5.57^b^	6.53^b^	6.80	73.47	8.17
HC	36.12^a^	116.20^a^	39.02^c^	0.99^e^	0.10^a^	6.35^b^	5.73^a^	7.14^a^	7.07	76.22	8.33
B	36.38^a^	116.55^a^	30.44^d^	2.30^c^	0.06^d^	6.43^a^	5.39^b^	6.38^b^	6.90	73.37	8.40
EC	35.37^a^	114.42^b^	47.35^a^	3.13^b^	0.07^c^	6.38^b^	5.48^b^	6.53^b^	6.95	73.43	8.16
Significance											
Storage (S)	<.0001**	<.0001**	<.0001**	<.0001**	<.0001**	<.0001**	<.0001**	<.0001**	<.0001**	<.0001**	n.s
Fertilizer (F)	<.0001**	<.0001**	<.0001**	<.0001**	<.0001**	<.0001**	.0124*	<.0001**	n.s	n.s	n.s
Post‐harvest (P)	<.0001**	<.0001**	<.0001**	<.0001**	<.0001**	<.0001**	<.0001**	<.0001**	<.0001**	<.0001**	<.0001**
S × F	<.0001**	n.s	n.s	n.s	.0004**	n.s	n.s	n.s	n.s	n.s	n.s
S × P	n.s	n.s	<.0001**	<.0001**	<.0001**	n.s	.0469*	n.s	n.s	n.s	n.s
F × P	n.s	n.s	n.s	n.s	n.s	n.s	n.s	n.s	n.s	n.s	n.s
S × F × P	n.s	n.s	n.s	n.s	n.s	n.s	n.s	n.s	n.s	n.s	n.s

*Note*: Data are represented as mean. The similar small letters indicate nonsignificant differences at a 5% level of probability using Duncan's test.

Abbreviations: Fertilizer treatments: AN, 560 kg·ha^−1^ ammonium nitrate (196 kg·ha^−1^ N); COM, compost + *Trichoderma harzianum* (196 kg·ha^−1^ N); CONTROL, no fertilizer; UREA_HIGH, 700 kg urea ha^−1^; UREA_LOW, 300 kg urea ha^−1^; UREA_OPT, 423 kg urea ha^−1^ (196 kg·ha^−1^ N); VER, vermicompost (196 kg·ha^−1^ N). Postharvest treatments: B, blanching in hot water; control, no postharvest treatment and packaging; EC, edible coating of psyllium seed mucilage; HC, hydrocooling; PP, plastic packaging (no treatment). AA, antioxidant activity; n.s, not significant; PPO, polyphenol oxidase activity; TP, total phenol; TSS, total soluble solids; Vit C. C, vitamin C content.

*p < .05; **p < .01.

### 3.2 Weight loss

**FIGURE 3:**
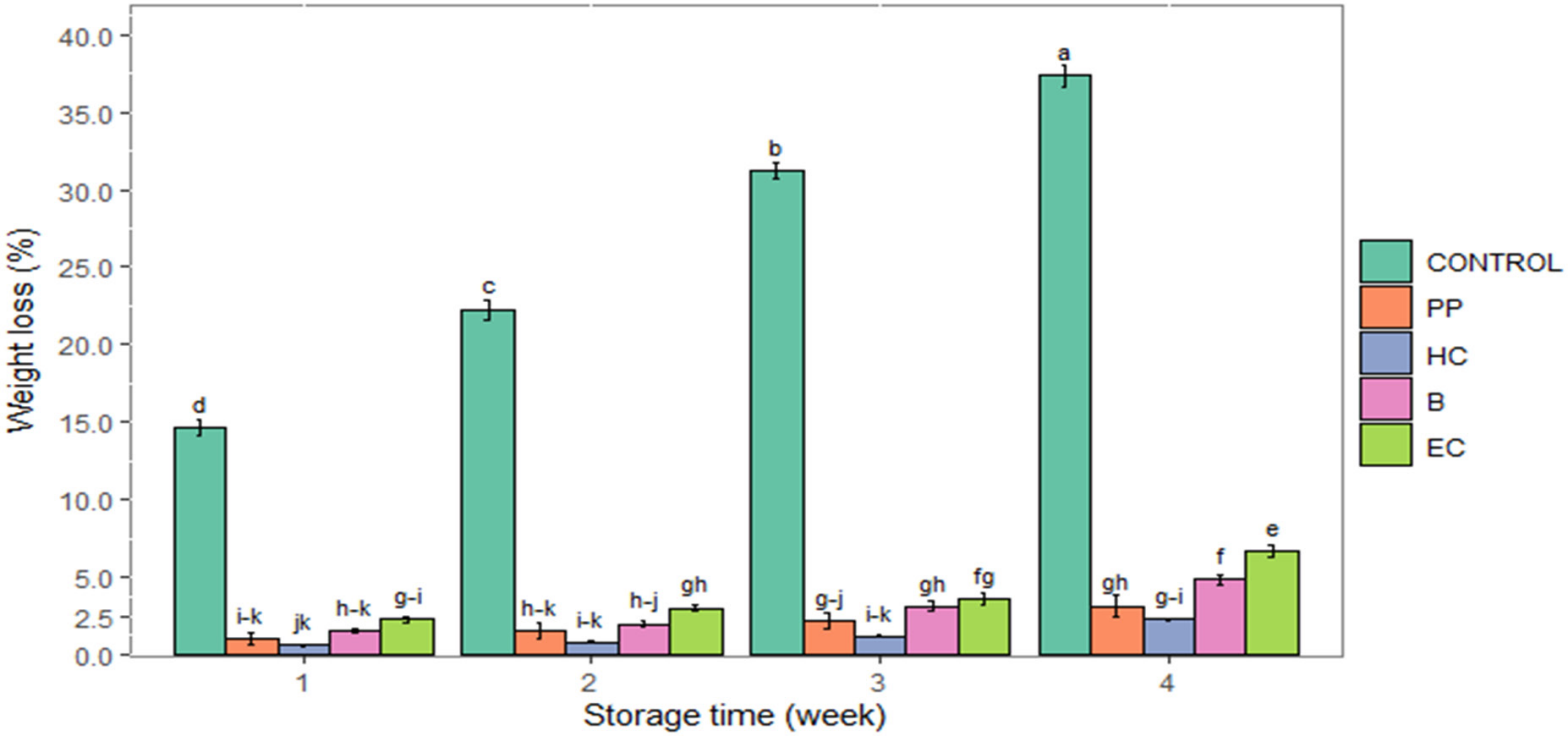
The interaction effect of storage time with postharvest treatments on weight loss in fresh‐cut celery during the storage (0–2°C, 85%–90% RH) for 4 weeks. B, blanching in hot water; CONTROL, no postharvest treatment and packaging; EC, edible coating of psyllium seed mucilage; HC, hydrocooling; PP, plastic packaging. Data are represented as mean. The similar small letters indicate non‐significant differences at a 5% level of probability using Duncan's test.

Interaction of storage time and postharvest treatment was found to be significant for weight loss (*p* ≤ .01) (Figure 3). The highest weight loss was in celery treated with UREA_HIGH (the highest rate of N fertilizer) and AN. The VER, COM, and control treatments had the lowest weight loss (Table 2). All postharvest treatments strongly prevented petiole weight loss during storage compared with the control. The lowest petiole weight loss was with HC (Figure 3).

### 3.3 TA, pH, and TSS

**FIGURE 4:**
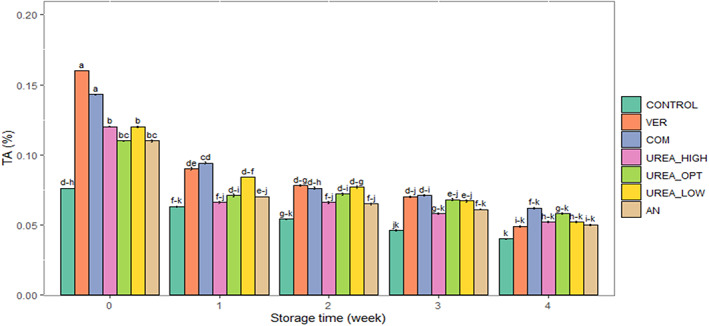
The interaction effect of storage time with nitrogen fertilizers (organic and chemical) on changes in titratable acidity (TA) in fresh‐cut celery during the storage (0–2°C, 85%–90% RH) for 4 weeks. AN, 560 kg ammonium nitrate ha^−1^ (196 kg·ha^−1^ N); COM, compost + *Trichoderma harzianum* (196 kg·ha^−1^ N); CONTROL, no fertilizer; UREA_HIGH, 700 kg urea ha^−1^ (324 kg·ha^−1^ N); UREA_LOW, 300 kg urea ha^−1^ (139 kg·ha^−1^ N); UREA_OPT, 423 kg urea ha^−1^ (196 kg·ha^−1^ N); VER, vermicompost (196 kg·ha^−1^ N). Data are represented as mean. The similar small letters indicate non‐significant differences at a 5% level of probability using Duncan's test.

**FIGURE 5:**
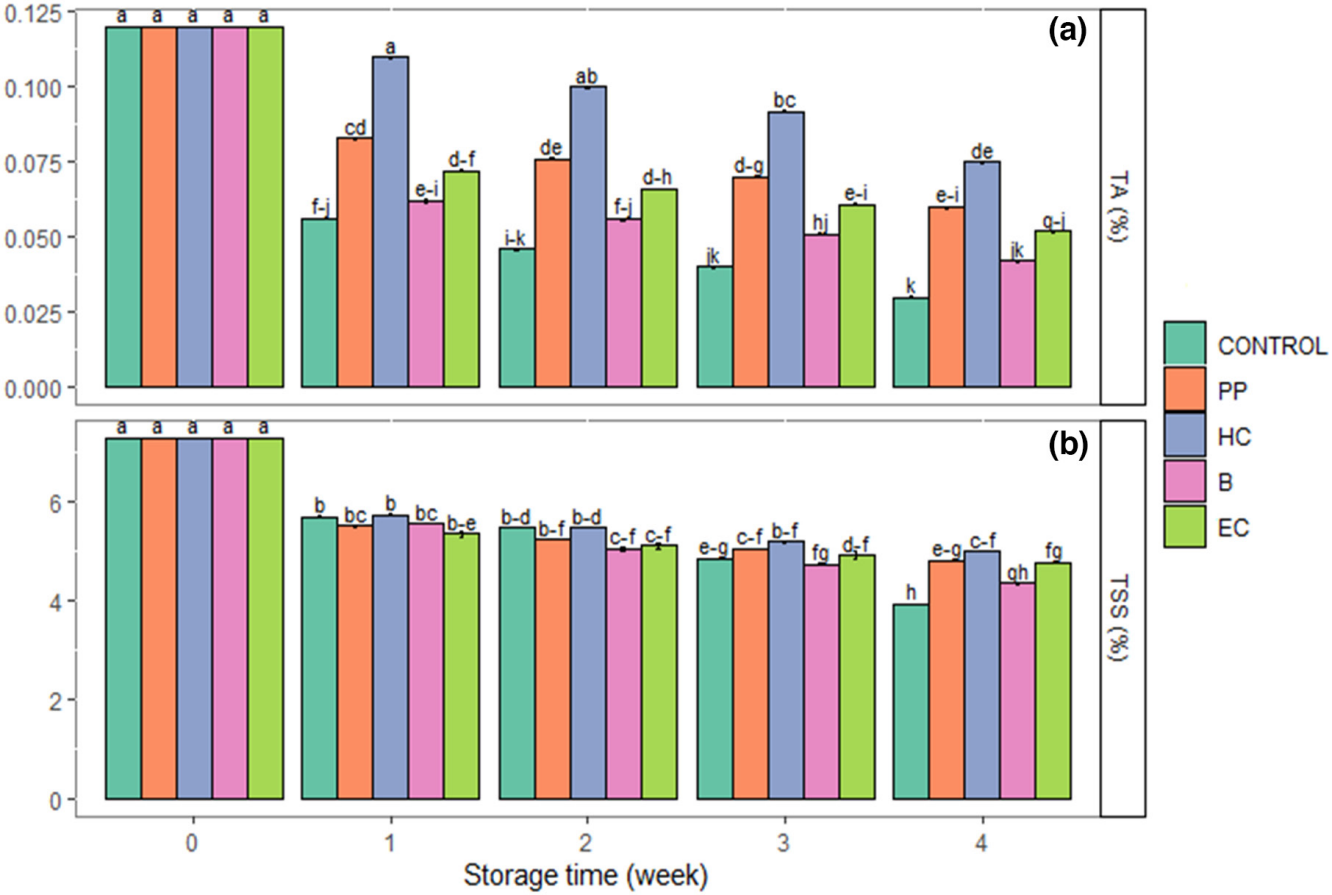
The interaction effect of storage time with postharvest treatments on changes in titratable acidity (a) and total soluble solids (b) in fresh‐cut celery during the storage (0–2°C, 85%–90% RH) for 4 weeks. B, blanching in hot water; CONTROL, no postharvest treatment and packaging; EC, edible coating of psyllium seed mucilage; HC, hydrocooling; PP, plastic packaging. Data are represented as mean. The similar small letters indicate non‐significant differences at a 5% level of probability using Duncan's test.

Interactions of storage time × fertilizer treatment were significant for TA and storage time × postharvest treatment for TA (*p* ≤ .01) and TSS (*p* < .05). Storage time, fertilizer, and postharvest treatments affected pH significantly (Table 2) (*p* ≤ .01). The TA and TSS decreased, and pH increased during storage. Among the fertilizer treatments, TA was highest in VER and COM at harvest time; at the end of storage, there was no significant difference between fertilizer treatments (Figure 4). The highest pH value was observed in the control and the lowest was in VER (Table 2). The TSS content was the highest with the organic fertilizers (VER and COM), followed by UREA_LOW (Table 2). The postharvest treatments PP, HC, and EC, reduced changes in TA (Figure 5A), while HC maintained TSS (Figure 5B) during storage. The PP, HC, and EC postharvest treatments had lower celery pH compared with B and control (Table 2).

### 3.4 Vitamin C

Vitamin C content in fresh‐cut celery was significantly affected by storage time, fertilizer treatments, and postharvest treatments (*p* ≤ .01) (Table 2). Vitamin C content decreased with increasing storage time (Table 2). The highest vitamin C content was observed with COM and the lowest with UREA_HIGH and AN (Table 2). Among the postharvest treatments, HC had the highest vitamin C content (Table 2).

### 3.5 TP, AA, and PPO activity

Storage time and fertilizer treatments significantly affected the TP and AA in celery (*p* ≤ .01). The PPO activity in celery decreased during storage (*p* ≤ .01) (Table 2). The postharvest treatments had no effect on TP, AA, and PPO activity. During the first week of storage, TP and AA increased, followed by a decline until the end of storage. A similar trend was observed in PPO activity (Table 2). Among fertilizer treatments, the highest TP levels were observed in COM and VER, followed by control, and the highest AA was obtained from COM (Table 2).

## 4 DISCUSSION

### 4.1 Color petiole (chrome, hue, and L*)

Consumers' perception of vegetable quality is influenced by the product's color and appearance. Changes in color may occur due to both preharvest and postharvest factors (Caracciolo et al., 2013; La Scalia et al., 2016; Miceli et al., 2019; Xiao et al., 2014). In the present study, the decrease in chroma during the storage suggests a decline in color saturation and an increase in the gray color components (Rizzo et al., 2011). A drop in chroma value in fresh‐cut celery during storage was also reported by Rizzo et al. (2011). A decrease in hue angle during the storage indicated changes in celery from green to yellowish‐green. These results are in accordance with Viña and Chaves (2003) and Rizzo et al. (2011), who reported that decreased hue angle during storage was accompanied by celery petioles changing from green to yellowish green. Gómez and Artés (2004) also reported that celery storage under air conditions decreased hue angle and increased celery yellowness. The breakdown of chlorophyll due to natural deterioration may result in the yellowing of fresh‐cut celery and increased L* value during storage of celery and lettuce (Kurubas et al., 2019; Viña & Chaves, 2003).

Organic fertilizers increased chroma in fresh‐cut celery at harvest time. The optimal celery harvesting time was reached earlier with organic fertilizers than with chemical fertilizers. Thus, a higher chroma value in the organic treatments may be due to accelerated maturation compared to the chemical treatments. Similarly, Navarro et al. (2020) reported that the chroma value in the organic fertilizer treatments was higher compared to the control. The organic fertilizer treatments delayed celery color changes the most (reduction in hue angle). Soil management techniques, particularly N fertilization, have been found to influence color in organic and inorganic systems. Zapata et al. (2013) reported that hue angle decreased progressively during broccoli storage, and the final hue angle in conventional broccoli was significantly lower than in organic broccoli. Navarro et al. (2020) also found the highest L* values in organic treatments compared to inorganic ones.

All the postharvest treatments maintained celery color. Chroma value and hue angle increased with the postharvest treatments compared to the control. The highest hue angle was in B, followed by HC and PP. Blanching possibly resulted in the inactivation of enzymes related to chlorophyll degradation, such as chlorophyllase (Xiao et al., 2012). Similar findings also revealed that hot water and hot air treatments delayed broccoli yellowing (Costa et al., 2006; Lemoine et al., 2009; Zapata et al., 2013). In the present study, postharvest treatments had lower L* values at the storage end than the control. These findings revealed that postharvest treatments might delay the increase of L* in fresh‐cut celery during storage. Viña et al. (2007) also stated a slight increase in the L* value of the fresh‐cut celery in control and hot air treatment during the entire storage. Kochhar and Kumar (2015) and Alibas and Koksal (2015) stated that precooling treatment slowed down and delayed the L* value changes in broccoli and cauliflower.

### 4.2 Weight loss

Horticultural crop weight loss leads to economic losses for growers, distributors, and storage operators (Kurubas et al., 2019). Postharvest weight loss of crops is related to their pre‐ and postharvest quality. Weight loss is one of the primary attributes affecting celery quality. Crops' weight loss may be due to respiration and transpiration losses after harvest (Kurubas et al., 2019). In the current study, the highest weight loss was obtained in celery grown with chemical fertilizers, probably because of the development of soft succulent tissues, with high moisture content, and with N chemical fertilizers (Tekeste et al., 2017). Bhattarai and Budathoki (2005) reported increased weight loss during storage in cauliflowers treated with chemical fertilizers and suggested losses may be due to the lack of the availability of micronutrients required for strengthening the cellular parts. Another study reported that excessive N fertilization increased weight loss in onion bulbs (Morsy et al., 2012; Tekalign et al., 2012). Lower weight loss of celeries grown with organic fertilizers compared with chemical ones could be attributed to increased and continuous uptake of nutrients from organic sources throughout the crop growth period (Tekeste et al., 2017).

Postharvest treatments declined the weight loss of fresh‐cut celery during storage compared with control. The HC treatment exhibited the lowest weight loss, probably because HC reduced celery temperature and thus decreased the difference in vapor pressure between the celery and the surrounding air (Senthilkumar et al., 2015). Plastic packaging proved an effective barrier to water vapor leading to decreased celery water loss (Fagundes et al., 2013). A weight loss of 2.5%–5% in fresh‐cut celery leads to wrinkling, shrinkage, and hollowing that may make celery undesirable for marketing (Avena‐Bustillos et al., 1997). Díaz‐Pérez (2019) reported that celery's maximum permissible weight loss is about 10%. In our study, at the end of storage, the weight loss of fresh‐cut celery in postharvest treatments ranged from 2.31% (HC) to 6.7% (EC), while the control showed excessive weight loss (37.36%). Edible coatings have been reported to reduce water loss in produce (Dong & Wang, 2017; Khodaei et al., 2021; Nasiri et al., 2018). Consistent with those reports, the EC treatment reduced celery weight loss. The mucilage in the coating possibly acted as a barrier to water vapor diffusion through the epidermis of celery petioles.

### 4.3 TA, pH, and TSS

Celery TA and TSS decreased during storage, possibly due to respiration utilizing organic reserves for energy production that is required to maintain metabolic activity. Increased pH during storage is likely due to the utilization of organic acids in respiratory activities (Budde et al., 2006). A high TA content in organic crops may help preserve the carbon–nitrogen ratio utilized for producing organic acids such as citric acid (the dominant acid in celery). Chemical fertilizers decreased celery TA and increased pH. Similar responses have been found by Zapata et al. (2013) in broccoli and Dabire et al. (2016) in tomatoes. The C/N balance theory can explain the increased TSS content in celery from organic fertilizers. When N availability is limited for plant growth, the metabolism shifts to generate more carbon‐containing compounds such as starch, cellulose, phenolics, and terpenoids (Brandt & Mølgaard, 2001; Bryant et al., 1983; Coley et al., 1985; Rembiałkowska, 2007).

The high TSS content has been observed in tomatoes, carrots, and onions (Bender et al., 2015; Kapoulas et al., 2011; Thangasamy et al., 2018). Mditshwa et al. (2017) stated that the relationship between high N and low TSS content in tomatoes could be attributed to excessive vegetative growth, which decreases the accumulation of sugars.

Among postharvest treatments, HC was the most effective in maintaining the TA. After harvesting, the deterioration rate is closely associated with the product's respiration rate. Since the respiration rate is affected by temperature, precooling by removing the product heat before storage reduces the respiration rate and diminishes the deterioration rate (Senthilkumar et al., 2015). These observations suggested that reduced metabolic activity probably prevented the consumption of organic acids in fresh‐cut celery. Shahi et al. (2012) and Akbudak et al. (2012) stated that precooling reduces metabolic activities, respiration rate, production of ethylene, and microbial activities and, thus, helps to preserve product quality and prolong its shelf life. During the storage, compared to the control, postharvest treatments (except B) maintained the pH by slowing down metabolic activities and reducing the utilization of metabolites. The EC and PP treatments reduced the gas exchange (water vapor and CO_2_ diffusion) between the celery and the surrounding environment (Rocculi et al., 2006; Saltveit, 2003). Hence, the consumption of respiratory substrates such as sugars and acids and, subsequently, the pH changes in fresh‐cut celery will be reduced. Among the postharvest treatments, the highest TSS content was recorded by HC treatment. Tian et al. (2016) reported that precooling reduced respiration and metabolic activity and subsequently delayed the decrease in TSS content in broccoli during storage.

### 4.4 Vitamin C

Vitamin C is one of the horticultural crops' essential nutritional quality attributes. In human diets, more than 90% of vitamin C is provided by fruits and vegetables (Lee & Kader, 2000). Vitamin C is the first defense mechanism against oxidative stress in plants (Gest et al., 2013). In our study, vitamin C content decreased with increasing time after harvest. Similarly, decreased vitamin C content during storage was found in broccoli (Carvalho & Clemente, 2004), fresh‐cut celery (Zhang et al., 2005), leafy vegetables (Konstantopoulou et al., 2010), and capsicum (Rahman et al., 2015).

In the present study, organic fertilizers improved vitamin C content compared with N chemical fertilizers. The relationship between vitamin C content and N nutrition varies among plant species. Studies show that the vitamin C content in organic crops was higher than in conventional crops (Magkos et al., 2003); our results are consistent with these findings. Vitamin C content in vegetables depends on many factors, such as plant nutrition, cultivar, cultivation practices, maturity, and light (Chebrolu et al., 2012). Differences in the nutrient compound of organic and chemical fertilizers and their effects on plant metabolism and soil ecology induce plants to synthesize non‐nitrogen‐containing compounds such as vitamin C. Excessive N fertilization decreases the level of vitamin C in crops. Reduced vitamin C content in chemical treatments could be due to a dilution effect, a response to increased vegetative growth because of excess plant‐available soil N (Chebrolu et al., 2012). In our study, HC was the most effective in maintaining vitamin C content among the postharvest treatments. The high TA content in HC treatment could act as a protective agent against the oxidation of ascorbic acid during storage (Irfan et al., 2013).

### 4.5 TP, AA, and PPO activity

An initial increase in TP during the storage may be due to the enhanced activity of the enzyme phenylalanine ammonia lyase (PAL) triggered by abiotic stress. The decline in TP later during storage is likely related to PPO's action that causes phenolic compounds' degradation. The initial rise of AA may reflect the increased activity of PAL, leading to a higher TP content. The decrease in AA after 7 days of storage may be because of decreased TP content due to the action of PPO (Mahn & Rubio, 2017). The changes in phenolic content during storage agreed with Amodio et al. (2014). The increase followed by the decrease in PPO activity during storage could be due to the changes in phenolic metabolism and the natural senescence in the minimal celery processing.

The high content of TP and AA in organic treatments may be caused by diverse macro‐ and micronutrients in organic fertilizers. In contrast, in the case of chemical fertilizers, only the N nutrient is available (Ibrahim et al., 2013). The high TP content in the control is likely related to the enhanced production of bioactive compounds and antioxidants as a preventive or protective agent against stresses, which may occur due to the low availability of macronutrients like N (Sharma et al., 2017). Barański et al. (2014) reported that the increased phenol content in organic lettuce might be due to environmental stress on plants that have not received synthetic fertilizers during development. Increased total phenols and antioxidants have also been reported with organic fertilization in lettuce (Kurubas et al., 2019), sweet pepper (Pérez‐López et al., 2007), chicory (Sinkovič et al., 2015), and tomato (Watanabe et al., 2015). Thus, organic fertilizers positively affected the production of total antioxidants and polyphenols in fresh‐cut celery.

## 5 CONCLUSIONS

This study evaluated the influence of nitrogen fertilizers (organic and chemical) and postharvest treatments on the visual and nutritional quality of fresh‐cut celery during storage. The results revealed that, compared to control, COM fertilizer led to improved visual quality such as chroma (38.88 vs. 30.80), hue (118.43 vs. 111.19), and L* (45.70 vs. 38.12), in addition to increased vitamin C content (12.6%), TP (7.9%), and AA (12.7%). It should be noted that among other fertilizers, COM also showed the highest effect in all the above‐mentioned parameters. VER fertilizer had the lowest celery weight loss (4.81% vs. 5.28%) and the highest TSS (6.02% vs. 5.03%). Among chemical fertilizers, UREA_LOW was a more effective treatment in maintaining celery color [chroma (37.16 vs. 30.8); hue (116.39 vs. 111.19); L* (42.24 vs. 38.12)] compared to control. Thus, high N rates with chemical fertilizers may be associated with poor celery quality.

Among postharvest treatments, compared to control, blanching better‐maintained celery color [chroma (36.38 vs. 33.17), hue (116.55 vs. 112.17), and L* value (30.44 vs. 48.59)] changes during storage. The lowest weight loss (0.99% vs. 21.12%) and the highest TA (0.1% vs. 0.05%), TSS (5.73% vs. 5.44%), and vitamin C contents (7.14 vs. 6.15) were obtained in HC postharvest treatment. Thus, B and HC positively affected fresh‐cut celery's visual and nutritional quality.

## CONFLICT OF INTEREST

The authors declare no conflict of interest in this study.
